# Does Iris Change Over Time?

**DOI:** 10.1371/journal.pone.0078333

**Published:** 2013-11-07

**Authors:** Hunny Mehrotra, Mayank Vatsa, Richa Singh, Banshidhar Majhi

**Affiliations:** 1 National Institute of Technology (NIT) Rourkela, Rourkela, India; 2 Indraprastha Institute of Information Technology (IIIT) Delhi, Delhi, India; Saitama Medical University, Japan

## Abstract

Iris as a biometric identifier is assumed to be stable over a period of time. However, some researchers have observed that for long time lapse, the genuine match score distribution shifts towards the impostor score distribution and the performance of iris recognition reduces. The main purpose of this study is to determine if the shift in genuine scores can be attributed to aging or not. The experiments are performed on the two publicly available iris aging databases namely, ND-Iris-Template-Aging-2008–2010 and ND-TimeLapseIris-2012 using a commercial matcher, VeriEye. While existing results are correct about increase in false rejection over time, we observe that it is primarily due to the presence of other covariates such as blur, noise, occlusion, and pupil dilation. This claim is substantiated with quality score comparison of the gallery and probe pairs.

## Introduction

Human growth or aging from newborn to toddler to adult to elderly is a natural phenomenon. This process leads to changes in different characteristics such as height, weight, face, gait, and voice. Several of these characteristics are being used as biometric identifiers. In literature, it is well established that over a long period of time, some biometric modalities such as face and voice can change, thereby reducing the recognition performance. On the other hand, iris is considered to be one of the most accurate and stable biometric modalities [1].

Daugman mentioned that *iris is well protected from the environment and stable over time* [1], [2]. This fact is also supported with the case study of Sharbat Gula, the Afghan girl whose iris templates were matched after the age difference of 18 years [3]. Owing to these characteristics of iris recognition, it is now used for authentication in several large scale government identification projects [4], [5]. However, recent research has claimed that iris recognition accuracy degrades over time [6]–[10]. Tome-Gonzalez et al. [6] studied the effect of time on the BiosecureID database with time lapse of maximum four months. The authors used Masek’s iris matcher [11] to investigate the effect of aging and analyzed that the intra-class variability increased over time with very little change in the impostor distribution. However, the time lapse considered for this study is very short (four months) and it is not justifiable to attribute aging to be the cause of performance reduction. Baker et al. [12] analyzed aging in iris recognition for multi-year time lapse. 6,797 iris images of 23 subjects were captured using the LG2200 iris camera. To evaluate the false non-match rate (FNMR) across time, images were collected from the same subjects first at an interval of less than 120 days and then at an interval of more than 1200 days. The images used in this study were manually screened for quality checks and the performance was evaluated using Neurotechnology VeriEye SDK [13] along with two other matchers. The authors inferred that factors such as pupil dilation, contact lens, occlusion, and sensor aging could not account for increase in false non match rates. Fairhurst et al. [14] studied aging on 79 users with 632 images. They modified Masek’s iris segmentation to reduce the segmentation errors and improve iris recognition accuracy. The authors concluded that dilation decreases with age thereby reducing the matching performance over time. Fenker and Bowyer [10], [15], [16] performed experiments with images pertaining to 322 subjects captured over a period of three years. They concluded that false non-match rate increases with time because of template aging. Ellavarason and Rathgeb [17] re-investigated the two year time lapse database used by Fenker and Bowyer [8] with six different iris feature extraction algorithms. They also observed that change in FNMR from short to long time lapse can be attributed to template aging. Sazonova et al. [18] examined the effect of elapsed time on iris recognition on 7628 images from 244 subjects acquired over a time lapse of two years at Clarkson University. The authors also considered the impact of quality factors such as local contrast, illumination, blur, and noise on the performance of iris recognition. VeriEye SDK and modified Masek’s algorithm were used for generating match scores and the significance of quality factors for recognition was also analyzed. They observed that the performance of both the matchers degrade with time. Recent research on aging by Czajka [19] used a dataset of 571 images collected from 58 eyes with up to eight years of time lapse acquired from 2003 to 2011. The results obtained using three different matchers and genuine scores exhibit template aging. The authors claimed that more accurate matchers are highly vulnerable to aging. Rankin et al. [9] performed another study for aging using visible spectrum images in which the images were acquired from both the eyes of 119 subjects. Even for a short time difference of six months, 32 out of 156 comparisons resulted in false rejections. This performance was obtained by applying both local and non-local operators. These error rates are very high compared to other studies. In response to Rankin et al. [9], Daugman and Downing [20] pointed out that their error rates were constant at all points in time studied, namely about 20%, showing no change in recognition accuracy over time. Recently, on two time-lapse private datasets collected by law enforcement agencies, using a complex regression analysis, National Institute of Standards and Technology (NIST) IREX report [21] suggests that *population-averaged recognition metrics are stable, consistent with the absence of iris ageing*.

It can be analyzed from the literature that researchers do not have a consensus on iris template aging. It is our assertion that proper analysis is required to understand the impact of aging on iris recognition. The objective of this study is to use the publicly available iris aging databases to understand iris aging and reasons for degradation in performance. In our experiments, it is observed that the increase in false rejection is due to poor acquisition, presence of occlusion, noise, and blur. The quality values of the falsely rejected gallery-probe pairs further substantiate the fact that the quality of iris images taken from two different sessions are different in comparison to the genuinely accepted pairs.

## Materials and Methods

This research re-investigates the challenge of iris template aging [6]–[10], [17]. The databases and algorithms used in this research are briefly explained below.

### Ethics Statements

All the experiments for this study are approved by the IIIT-Delhi Ethics Board. The iris databases are obtained from the CVRL Lab, University of Notre Dame [22], which are prepared as per the UND IRB guidelines with written consent obtained from the participants.

### Databases

Two publicly available iris databases are used to investigate the effect of aging on iris recognition with a time lapse of two years and four years.

1. ND-Iris-Template-Aging-2008–2010 Database: The images in the ND-Iris-Template-Aging-2008–2010 database [10] are acquired using the LG 4000 iris sensor during spring 2008, spring 2009, and spring 2010. This allows to conduct two different one year template aging studies, i.e., for the year 2008–2009 and 2009–2010, and one two year template aging study for 2008–2010. The number of subjects in the study are 88, 157, and 40 for 2008–2009, 2009–2010, and 2008–2010 sessions respectively.
2. ND-TimeLapseIris-2012 Database: The ND-TimeLapseIris-2012 database [12] contains images acquired with the LG2200 iris camera located in the same studio throughout all the acquisitions. A total of 6797 images are collected from 23 subjects (46 irises) in between 2004 to 2008. The age of these subjects ranges from 22 to 56 years where 16 subjects are male and 7 are female.

### Commercial Matcher

Iris recognition is performed using the commercial VeriEye SDK [13], that has shown good performance in the state-of-art evaluations by NIST [23]. VeriEye contains advanced segmentation, enrollment, and matching routines. For segmentation, VeriEye uses active shape models that accurately detect contours of the irises which are not perfect circles. The enrollment and matching routines are fast and yield very high matching performance/accuracy.

### Experimental Protocol

The experimental protocol used to perform the experiments are explained below for each database.

1. ND-Iris-Template-Aging-2008–2010: The protocol followed for this database is same as provided by Fenker and Bowyer [10]. All the possible genuine comparisons are provided as part of the protocol. In the experiments, *short* refers to images captured within the same year whereas *long* refers to comparisons across years. The cross session irises for this particular study refers to the images captured over a time lapse of one or two years.
2. ND-TimeLapseIris-2012: The protocol followed for this study consists of two sets of image pairs [12]. The *short* time lapse set consists of image pairs with no more than 120 days of time lapse between them. The *long* time lapse set consists of image pairs with more than 1200 days of time lapse. An image instance can participate in multiple short and long time lapse pairs. Each image instance has several associated attributes such as date of acquisition, unit, color, glasses, and contact lens. For a genuine comparison, the units of two iris images must match along with the time lapse mentioned above. However, in the experiments, some false acceptance cases with exceptionally high scores (almost close to genuine acceptance) were observed. On carefully analyzing these images, we observed that there are ground truth errors in the database due to incorrect ID labels. These incorrectly labeled instances belong to ids: 04870d1810 and 04888d395. The cases associated with these incorrectly labeled ids were not considered in this study.

## Results

If the performance degradation is caused due to aging, then this should hold true for all genuine comparisons pertaining to an individual across different sessions. Therefore, three sets of experiments are performed to closely study the cause of rejections that happen over time. The detailed description and analysis of each experiment is given below.

### Experiment 1: Performance Evaluation

**Figure 1:**
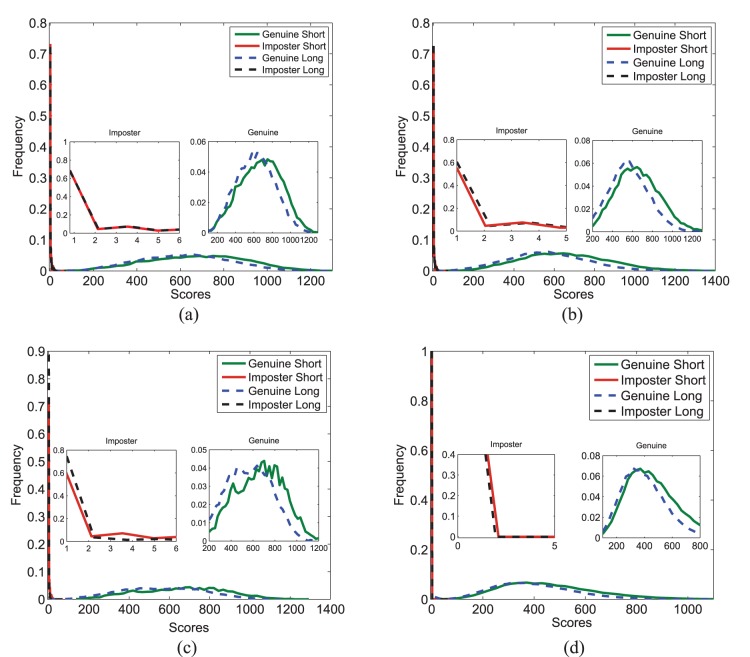
Histogram plots for experiment 1. Time lapse (a) 2008–2009, (b) 2009–2010, (c) 2008–2010 on ND-Iris-Template-Aging-2008–2010 database, and (d) 2004–2008 on ND-TimeLapseIris-2012 database.

**Figure 2:**
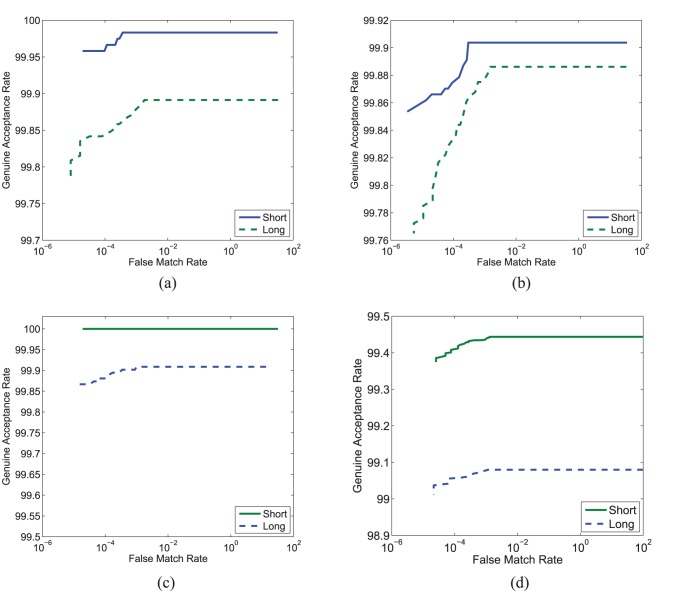
ROC curves for experiment 1. Time lapse (a) 2008–2009, (b) 2009–2010, (c) 2008–2010 on ND-Iris-Template-Aging-2008–2010 database, and (d) 2004–2008 on ND-TimeLapseIris-2012 database.

The first experiment is performed to compute iris matching accuracy for both short and long time lapses. Genuine and impostor scores are obtained using the VeriEye SDK on the protocols explained earlier. Table 1 shows the genuine accept rate (GAR) at 0.001% false match rate (FMR) for both long and short time lapses on the ND-Iris-Template-Aging-2008–2010 and ND-TimeLapseIris-2012 databases. The results show that we are able to reproduce the accuracies reported by the original papers. The distribution of genuine and impostor scores are shown in Figure 1. There is no evident shift in the impostor scores whereas the genuine scores show a shift towards the impostor scores for long time lapse. Further, the receiver operating characteristic (ROC) curves in Figure 2 show a slight variation between long and short time lapses. The performance with long time lapse is slightly lower than the short time lapse. McNemar test [24] shows that at 95% confidence interval, these results are statistically significant. This experiment shows that there is a reduction in the verification results in the long time lapse. However, the cause of shift in distributions or decrement in genuine accept rate cannot merely be attributed to aging. Therefore, the next experiments focus on determining the cause for performance reduction.

**Table 1:** Verification results for experiments 1 and 2 on the two databases using VeriEye [13].

Database	Time lapse	Experiment 1	Experiment 2	
		GAR (%)	GA	FR
ND-Iris-Template Aging-2008–2010	2008–2009 (Short)	99.96	5,434	0
ND-Iris-Template Aging-2008–2010	2008–2009 (Long)	99.88	14,202	17
ND-Iris-Template Aging-2008–2010	2009–2010 (Short)	99.90	6,720	4
ND-Iris-Template Aging-2008–2010	2009–2010 (Long)	99.88	15,230	28
ND-Iris-Template Aging-2008–2010	2008–2010 (Short)	100.00	5,434	0
ND-Iris-Template Aging-2008–2010	2008–2010 (Long)	99.90	13,425	19
ND-TimeLapseIris-2012	Short	99.44	128,690	815
ND-TimeLapseIris-2012	Long	99.08	128,875	1,280

The GAR is computed at 0.001% FMR. (GA and FR represent genuine accept and false reject respectively.

### Experiment 2: Common Subjects Over Time

It is our hypothesis that for a given subject, if aging exists and if the false rejections can be attributed to aging, then all the iris images of this subject with the same or more time lapse should be rejected. With this hypothesis, we analyze false rejection cases to understand if the rejections are occurring due to aging or any other factor. In the ND-Iris-Template-Aging-2008–2010 database, the subjects that are common over multiple years are selected. There are 34 subjects common to 2008, 2009, and 2010 sessions. These common subjects are chosen to carefully study the cases of rejection and investigate the corresponding cases which are otherwise accepted. Table 1 illustrates the total number of genuine comparisons pertaining to these 34 subjects along with the number of false rejects. Here, all the experiments are performed using a threshold that produces the FMR of 0% in order to solely concentrate on the cause of genuine rejections over a period of time. Similarly, the rejections at 0% FMR from the ND-TimeLapseIris-2012 database are also obtained (all 23 subjects are present in both short and long time lapses). The number of genuine matches and false rejections at 0% FMR are shown in Table 1.

**Figure 3:**
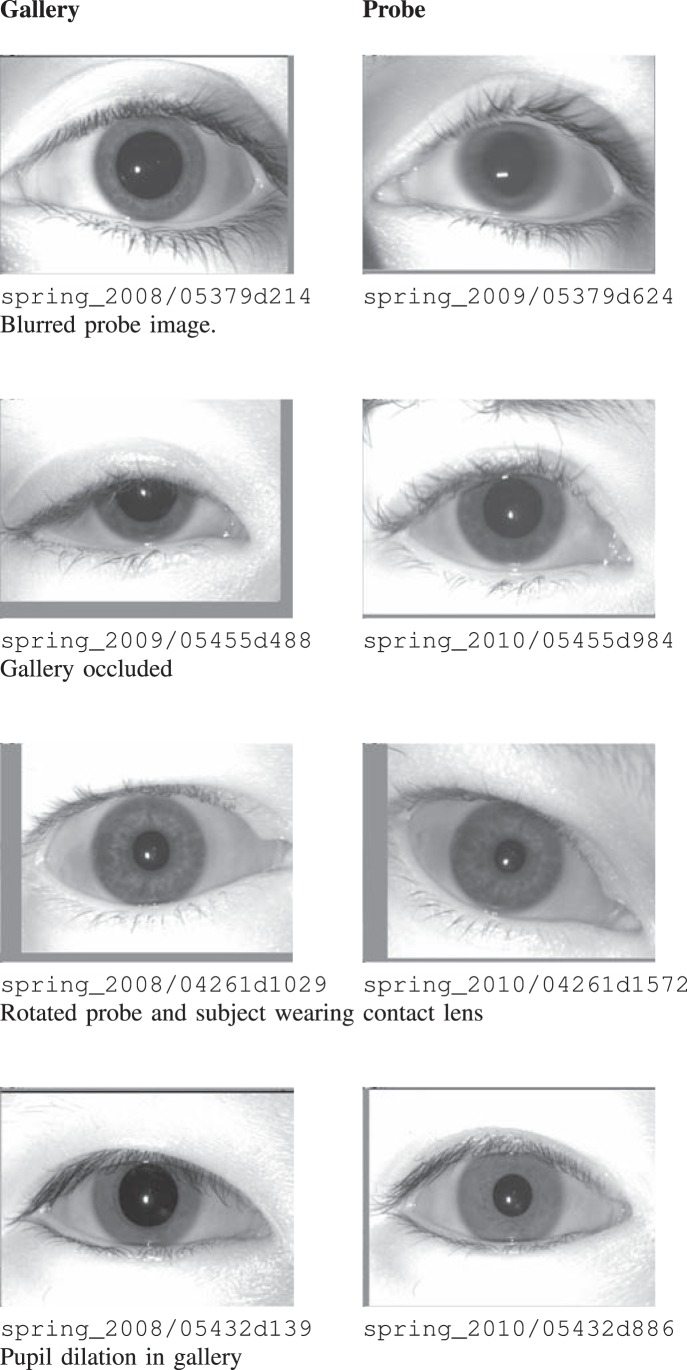
Cases of false non-match for variation in time on the ND-Iris-Template-Aging- 2008–2010 database. Here, the gallery and probe instances are taken from cross sessions and the possible cause of rejection is mentioned as a remark. The image labels are provided for reproducibility.

**Figure 4:**
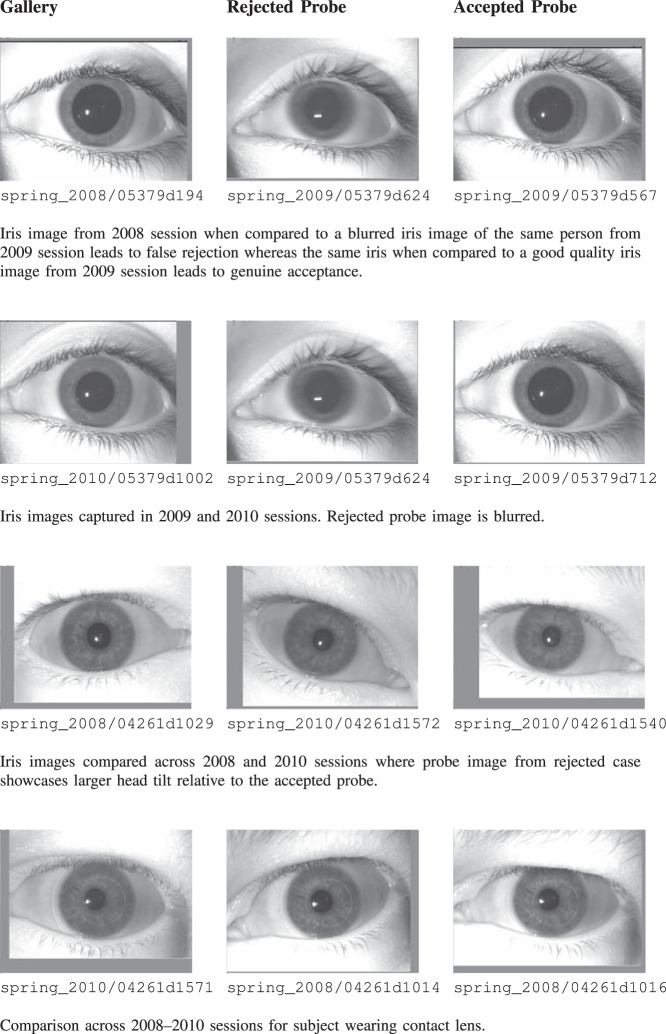
Illustrating cross session iris comparisons for the ND-Iris-Template-Aging- 2008–2010 database. The gallery instance (1st column) is compared to probe images (columns 2 and 3) that belong to the same session. While one probe is rejected, the other probe image for the same session is accepted. The cause of rejection is stated as remark below the images. These examples illustrate that aging is not the key factor in performance degradation on this database rather other factors affected the recognizability.

**Figure 5:**
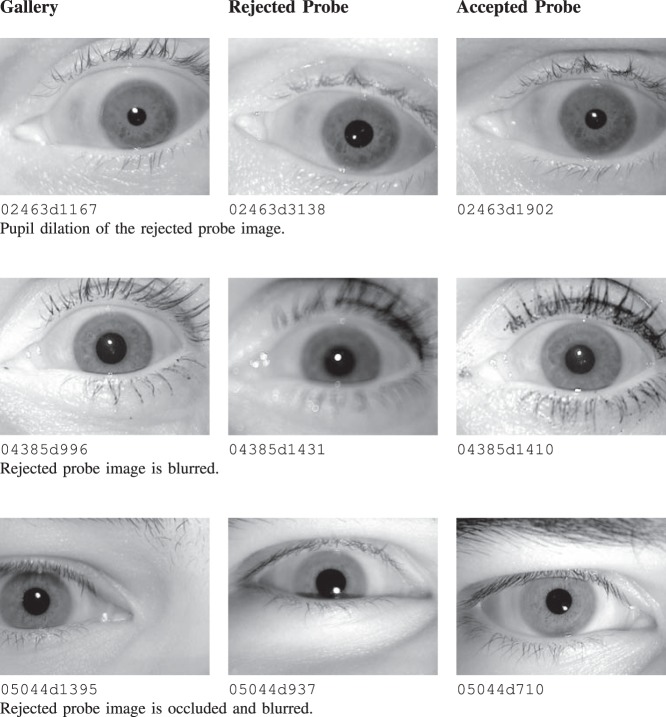
Cross session iris comparisons for the ND-TimeLapseIris-2012 database. The accepted and rejected probes belong to the same session.

- Figure 3 illustrates sample cases of false rejection on the ND-Iris-Template-Aging-2008–2010 database. The images in this database are labeled as session_year/instance_id where instance_id contains subject id as the first five characters followed by the iris instance number. It is interesting to note that for time lapse 2008–2009 (Long), all the false rejections are caused due to a single probe instance (spring_2009/05379d624) which is actually blurred. The same instance when compared with other irises in 2009, for short comparison, also leads to rejections. For 2009–2010 (Long), 28 false rejections are observed which is the maximum in any year. These cases are also studied in detail and after careful investigation, it is found that all the rejections are either due to blurring, occlusion, off-angle, or pupil dilation.
- For two year time lapse, i.e., 2008–2010 (Long), there are 19 false rejections. It is observed that these rejections are also due to noisy gallery or noisy probe instances. Similarly, as shown in Table 1, there are 1280 cases of false rejection for long time lapse in the ND-TimeLapseIris-2012 database. This number is actually very small compared to the total number of genuine matches, i.e., 128,875. Here also, it is observed that the cases are rejected primarily due to variations in quality (quality aspect is discussed as part of Experiment 3).
- Figures 4 and 5 show cases from the gallery image captured in one session and probe images captured in session from another year. It is observed that some probe images of the subject match whereas others from the same session and same subject do not match. Thus, it can be inferred that aging is not the cause of false rejections and there are other covariates/challenges involved.
- The test of proportions at 95% confidence interval, where proportions 
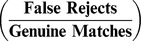
 are calculated between one year, two year, and four year differences, also show that the proportions are statistically *non-significant*.

### Experiment 3: Analyzing Quality of Rejected Iris Pairs

From experiment 2, it can be inferred that the performance reduction on the ND-Iris-Template-Aging-2008–2010 and ND-TimeLapseIris-2012 databases is not due to iris template aging. Therefore, to determine the actual cause of degradation, we analyze the image quality of the gallery and probe pairs. The quality of iris images is assessed using the quality assessment algorithm proposed by Kalka *et al.* [25]. It computes quality metrics such as blur, rotation, off-angle, and occlusion to determine a single composite quality score. The quality values of the gallery and probe images are obtained for the falsely rejected and the corresponding genuinely accepted pairs of these subjects over long time lapse. Let 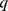 be the quality of an input iris image. For a gallery and probe iris image pair 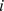, the absolute difference, 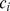, is calculated as 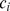 = 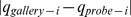. This absolute difference is calculated for all the selected genuine accept and false reject cases and 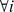, 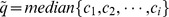 is obtained. Table 2 illustrates the median quality differences for the examined datasets. It can be observed that 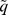 for falsely rejected pairs is higher than genuinely accepted iris pairs. This observation suggests that the pairs are falsely rejected because of the increased difference in the quality of gallery and probe image pairs.

**Table 2:** Difference between the quality scores of the gallery and probe pairs (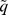) for experiment 3.

Database	Time lapse	Quality Difference (Median)	
		Genuine Accepts	False Rejects
ND-Iris-Template-Aging-2008–2010	2008–2009 (Long)	0.17	0.46
ND-Iris-Template-Aging-2008–2010	2009–2010 (Long)	0.26	0.36
ND-Iris-Template-Aging-2008–2010	2008–2010 (Long)	0.14	0.27
ND-TimeLapseIris-2012	Long	0.12	0.16

The results of these three experiments put together suggest that the false rejections on the two iris databases are mainly due to occlusion, rotation, blurring, illumination and pupil dilation or constriction.

## Discussion and Conclusion

Recent research results initiated the discussion on whether aging affects iris templates or not. While some researchers support that aging affects the performance, others are of the opinion that it does not have a prominent effect. Using publicly available iris template aging databases, this paper shows that the reduced performance of iris recognition may not be caused by aging but due to noise and differences in the quality of gallery and probe pairs. Some of our observations are:

- Though, for long time lapse, genuine score distributions demonstrate a shift towards the impostor score distributions, empirical investigation suggests that the rejections are caused by improper capture that leads to occlusion, rotation, blurring, illumination, and pupil dilation or constriction in iris images.
- The analysis also suggests that had aging been the cause of rejections then this should uniformly affect the performance. However, only few samples with time difference are rejected and other samples of the same subject with similar time difference are accepted.
- Existing literature suggests that one of the factors for template aging is pupil dilation-constriction with human growth. While there are reported results in medical literature to support this claim, it is more prevalent in elderly people only. In order to analyze this effect, we should collect iris images of different individuals at 4–10 years apart, specially for people with age of over 50 years.

It is our assertion that iris template aging is an important research problem which requires a longitudinal study; similar to face biometrics where 2–60 years time lapse has been studied. We believe that to conduct a proper study on longitudinal effects, an ideal approach would be to collect a controlled iris database of individuals in different age groups over a period of several years. Such a database can help in understanding the factors that may affect iris recognition performance such as sensor aging, interoperability, human growth (pupil dilation-constriction), and image quality.
